# HIV‐associated morbidity and mortality in a setting of high ART coverage: prospective surveillance results from a district hospital in Botswana

**DOI:** 10.1002/jia2.25428

**Published:** 2019-12-18

**Authors:** Tomer Barak, Dayna T Neo, Neo Tapela, Patricia Mophuthegi, Rebbeca Zash, Ketenga Kalenga, Melissa EO Perry, Mompati Malane, Joseph Makhema, Shahin Lockman, Roger Shapiro

**Affiliations:** ^1^ Botswana‐Harvard AIDS Institute Partnership Gaborone Botswana; ^2^ Department of Medicine Beth Israel Deaconess Medical Center Boston MA USA; ^3^ Department of Medicine Scottish Livingstone Hospital Molepolole Botswana; ^4^ Department of Obstetrics and Gynecology Beth Israel Deaconess Medical Center Boston MA USA; ^5^ Nuffield Department of Population Health University of Oxford Oxford United Kingdom; ^6^ Division of Global Health & Equity Brigham & Women's Hospital Boston MA USA; ^7^ Division of Infectious Disease Beth Israel Deaconess Medical Center Boston MA USA; ^8^ Department of Genitourinary Medicine Western Health and Social Care Trust Londonderry United Kingdom; ^9^ Department of Immunology and Infectious Diseases Harvard T.H. Chan School of Public Health Boston MA USA

**Keywords:** HIV, mortality, inpatient, hospital, Botswana, Africa

## Abstract

**Introduction:**

Antiretroviral therapy (ART) has significantly improved survival in Africa in recent years. In Botswana, where adult HIV prevalence is 21.9%, AIDS‐related mortality is estimated to have declined by 58% between 2005 and 2013 following the initial wide‐spread introduction of ART, and ART coverage has steadily increased reaching 84% in 2016. However, there remains little data about the burden of HIV and its impact on mortality in the hospital setting where most deaths occur. We aimed to describe the burden of HIV and related morbidity and mortality among hospitalized medical patients in a district hospital in southern Botswana in the era of widespread ART coverage.

**Methods:**

We prospectively reviewed medical admissions to Scottish Livingstone Hospital from December 2015 to November 2017 and recorded HIV status, demographics, clinical characteristics and final diagnoses at discharge, death or transfer. We ascertained outcomes and determined factors associated with mortality. Results were compared with similar surveillance data collected at the same facility in 2011 to 2012.

**Results:**

A total of 2316 admissions occurred involving 1969 patients; 42.4% were of HIV‐positive patients, 46.9% of HIV‐negative patients and 10.7% of patients with unknown HIV status. Compared to HIV‐negative patients, HIV‐positive patients had younger age (mean 42 vs. 64 years, *p* < 0.0001) and higher mortality (22.2% vs. 18.0%, *p* = 0.03). Tuberculosis was the leading diagnosis among mortality cases in both groups but accounted for a higher proportion of deaths among HIV‐positive admissions (44.5%) compared with HIV‐negative admissions (19.4%, *p* < 0.0001). Compared with similar surveillance in 2011 to 2012, HIV prevalence was lower (42.4% vs. 47.6%, *p* < 0.01), and among HIV‐positive admissions: ART coverage was higher (72.2% vs. 56.2%, *p* < 0.0001), viral load suppression was similar (78.6% vs. 80.3%, *p* = 0.77), CD4 counts were higher (55.0% vs. 44.6% with CD4 ≥200 cells/mm^3^, *p* < 0.001), mortality was similar (22.2 vs. 22.7%, *p* = 0.93), tuberculosis diagnoses increased (27.5% vs. 20.1%, *p* < 0.01) and tuberculosis‐associated mortality was higher (35.9% vs. 24.7%, *p* = 0.05).

**Conclusions:**

Despite high ART‐coverage in Botswana, HIV‐positive patients continue to be disproportionately represented among hospital admissions and deaths. Deaths from tuberculosis may be contributing to lack of reduction in inpatient mortality. Our findings suggest that control of HIV and tuberculosis remain top priorities for reducing inpatient mortality in Botswana.

## 1 Introduction

The introduction and scale‐up of antiretroviral therapy (ART) over the past decade has led to significant improvements in quality of life, life expectancy and mortality in sub‐Saharan Africa, but the impact of expanded ART availability on the burden of HIV‐related mortality in the hospital setting remains unknown 1, 2. Recent studies from countries in sub‐Saharan Africa report high inpatient mortality rates despite increased access to free ART 3, 4, 5, 6, 7, 8. In‐hospital mortality among HIV‐positive patients may be influenced by factors such as rate and timing of ART uptake, toxicity of available ART regimens, co‐morbidities and facility level and health system resources in different settings. The persistently high inpatient mortality reported across diverse settings in Africa suggests that some HIV‐related conditions may not be addressed (or immediately addressed) following ART initiation, and that the scale‐up of ART programmes has not in itself been sufficient to curb the impact of HIV‐related mortality on hospital systems.

Botswana, a middle‐income country in southern Africa, has the world's third highest prevalence of HIV with a current adult HIV prevalence of 21.9% 9. Botswana has long been considered a leader in HIV response amongst African nations and in 2001 became the first country in sub‐Saharan Africa to provide free ART. This coverage is now offered regardless of CD4 cell count and reaches approximately 84% of citizens living with HIV 9.As a consequence, model‐based trend estimations suggest that AIDS‐related mortality in Botswana has declined by 59% between 2005 and 2013 9, 10 and by a further 31% between 2013 and 2018 11. However, there remains little ART‐era data from Botswana evaluating the burden of HIV‐related mortality in the hospital setting, where most deaths occur.

We established comprehensive inpatient data surveillance at Scottish Livingstone Hospital (SLH), a government district hospital in southern Botswana. Our objectives were to assess the burden of HIV among medical inpatients at SLH, to describe mortality by HIV status, and to identify factors independently associated with inpatient mortality.

## 2 Methods

### 2.1 Study design

We conducted a prospective review of all patients discharged from the adult medical wards at SLH over a 24‐month period from 1 December 2015 to 30 November 2017. All medical admissions to the adult female and male medical wards were included. As per hospital policy, adult admissions included females ≥12 years and males ≥13 years.

### 2.2 Study site

SLH is a 350‐bed public sector district hospital located in Molepolole (population 73,102), the largest village in Botswana and the main referral centre for the Kweneng East district, which has a population of approximately 190,000 and an overall HIV prevalence of 19% 12, 13. ART is offered in 20 public sector clinics across the district and, though exact ART‐coverage data from the district is unavailable, it is estimated to approximate the national average (UNAIDS, private communication).

Available diagnostic resources at SLH include basic haematology, chemistry and microbiology testing, plain x‐rays and ultrasound. Stock‐outs of reagents and machine breakdowns frequently interfere with regular availability of diagnostic test results. The hospital's medical wards are staffed by non‐specialized medical officers, rotating medical interns and internal medicine specialists.

### 2.3 Data collection

Collected data included patient demographics, co‐morbidities, HIV status, HIV testing results, anti‐retroviral treatment, CD4 count, HIV viral load, final diagnosis at discharge, death or transfer and mortality status at discharge or following transfer. Data were collected upon patient discharge, transfer or death and entered into an electronic data abstraction form. Clinical data were collected from patients' hand‐written medical records. Laboratory data were collected from the Integrated Patient Management System (IPMS), an electronic medical record system used in most hospitals in Botswana. Post‐transfer outcomes were ascertained from IPMS and, when not available electronically, by direct communication with the accepting facility. Data were collected by trained research assistants and medical ward doctors, and all final discharge diagnoses underwent secondary review and adjudication by an internal medicine specialist.

### 2.4 Statistical methods

Final outcomes were classified as “discharged from hospital alive,” “died in hospital,” “left against medical advice” or “transferred to another ward of facility.” For the purposes of final outcome analysis, patients who left against medical advice were considered alive at final discharge and transferred patients were analysed per the hospitalization outcome at the location to which they were transferred.

All data were analysed using SAS 9.4 (SAS Institute, Cary, NC). We compared categorical data using chi‐square and Fisher's exact tests and t‐tests or Wilcoxon rank‐sum tests were used to compare continuous variables. Risk factors for mortality were evaluated using modified Poisson regression to estimate risk ratios and 95% confidence intervals, adjusted for age, sex and presence or absence of known co‐morbidities.

Approval to conduct data collection and report outcomes were granted by institutional review boards at Scottish Livingstone Hospital, Molepolole, Botswana and Beth Israel Deaconess Medical Center, Boston, US and by the Botswana Human Research and Development Committee, Gaborone, Botswana.

## 3 Results

### 3.1 Study population

Over the 24‐month surveillance period, 2316 admissions occurred among 1969 patients; 1079 (46.6%) admissions were to the male medical ward, and 1237 (53.4%) were to the female medical ward. Median patient age at admission was 51 years (interquartile range (IQR): 34 to 71), and the median duration of admission was 6.0 days (IQR: 3.0 to 11.0). The majority of admissions were among patients who lived in Molepolole (66.7%), with a smaller number of patients admitted from rural villages in the Kweneng East district (25.3%), other rural areas (3.4%), or from the nearby urban centre of Gaborone (2.6%). (Table 1).

**Table 1:** Baseline characteristics of medical admissions stratified by final HIV status^a^

Admission characteristics	HIV+	HIV−	HIV unknown	Total
HIV status on admission	904 (39.0)	575 (24.8)	837 (36.1)	2316 (100.0)
Final HIV status	**983 (42.4)**	**1086 (46.9)**	**247 (10.7)**	**2316 (100.0)**
Age (years)	42 (33 to 52)	64 (40 to 78)	68 (24 to 81)	51 (34 to 71)
Sex				
Male	478 (48.6)	507 (46.7)	94 (38.1)	1079 (46.6)
Female	505 (51.4)	579 (53.3)	153 (61.9)	1237 (53.4)
Admission type during study period				
First admission	817 (83.1)	918 (84.5)	234 (94.7)	1969 (85.0)
Repeat admission	166 (16.9)	168 (15.5)	13 (5.3)	347 (15.0)
Current residence				
Molepolole	645 (65.6)	729 (67.1)	171 (69.2)	1545 (66.7)
Kweneng East	243 (24.7)	285(26.2)	58 (23.5)	586 (25.3)
Other rural village	36 (3.7)	33 (3.0)	9 (3.6)	78 (3.4)
Gaborone (Urban)	34 (3.5)	23 (2.1)	4 (1.6)	61 (2.6)
Unknown	25 (25.4)	16 (14.7)	5 (2.0)	46 (2.0)
Co‐morbidities				
Hypertension	140 (14.2)	527 (48.5)	110 (445)	777 (33.6)
Diabetes	78 (7.9)	264 (24.3)	50 (20.2)	392 (16.9)
Heart disease	44 (4.5)	167 (15.4)	28 (11.3)	239 (10.3)
Lung disease	80 (8.1)	147 (13.5)	24 (9.7)	251 (10.8)
History of tuberculosis	291 (29.6)	144 (3.3)	25 (10.1)	460 (19.9)
Any comorbidity^b^	353 (35.9)	814 (75.0)	166 (67.2)	1333 (57.6)

a Data presented as median (interquartile range) or n (%)

b includes co‐morbidities listed above and other co‐morbidities

### 3.2 HIV status and HIV testing

A total of 904 (39.0%) admissions were of patients known to be HIV‐positive prior to presentation, 575 (24.8%) were of patients with a previously negative test and 837 (36.1%) were of patients with unknown HIV status. Among patient admissions with unknown HIV status on presentation, 591 (70.6%) were tested during the hospitalization and of those tested 69 (11.7%) were found to be HIV positive. Males were as likely as females to have an unknown HIV status prior to admission (35.8% of male admission vs. 36.5% of female admissions, *p* = 0.73) and among those who were tested during hospitalization the risk of HIV positivity did not differ significantly among male admissions compared with female admissions (13.7% vs. 9.7%, *p* = 0.13). Among patient admissions with prior documentation of a negative HIV test, 157 (27.3%) re‐tests occurred during the hospitalization and 10 (6.4%) were found to be HIV positive.

In total, there were 983 (42.4%) admissions with a final HIV‐positive status, 1086 (46.9%) with a final HIV‐negative status, and 247 (10.7%) with a final unknown HIV status. Overall, 48.6% of HIV‐positive admissions were male and 51.4% female. (Table 1).

### 3.3 Baseline characteristics by final HIV status

Among all admissions with a known final HIV status, differences in the number of male and female admissions by HIV status were not observed (*p* = 0.38). In addition, geographic distribution was similar between HIV‐positive and HIV‐negative admissions. However, HIV‐positive admissions were younger, with a median age at admission of 42 years (IQR 33 to 52) compared with 64 years (IQR 40 to 78) (*p* < 0.0001) for HIV‐negative admissions and had fewer recorded chronic co‐morbidities compared with HIV‐negative admissions (35.9% vs. 75.0%, *p* < 0.0001). Baseline characteristics by HIV status are outlined in Table 1.

### 3.4 ART, CD4, HIV viral load

Among admissions of patients who were known to be HIV positive prior to admission and whose pre‐admission ART status was known, 653(72.5%) were on ART. Males were more likely not to have been on ART prior to admission compared with females (32.4% vs. 23.0%, *p* = 0.002). Among admissions of patients on ART who had a known viral load, 493 (89.6%) had undetectable viral load (<400 cp/mL) at their last test (either prior to or during the admission). Detectable viral loads (≥400 cp/mL), despite being on ART, were more likely to occur among younger patients, with a median age of 37 years (IQR: 31 to 49) compared with a median age of 43 years (IQR: 36 to 55) in patients who had undetectable viral loads (*p* = 0.02). The occurrence of detectable viral loads in patients on ART did not differ significantly between males and females (7.9 vs. 9.4%; *p* = 0.48). Being on ART prior to admission was associated with higher CD4 cell count at last test (either prior to or during admission), with a median of 310 cells/mL (IQR, 136 to 522) for those on ART versus 103 cells/mL (IQR, 40 to 252) for those not on ART, *p* < 0.0001.

### 3.5 Admission outcomes and effect of HIV‐positive status on mortality

In total, 420 (18.1%) admissions resulted in death in‐hospital, 1749 (75.5%) resulted in live discharge, 17 (0.7%) resulted in patient leaving against medical advice, and 130 (5.6%) resulted in patient transfer to another ward or facility. Among patient transfers, 42 (32.3%) resulted in patient death, 87 (66.9%) resulted in live discharge from the ward or facility the patient was transferred to, and in 1 (0.8%) the post‐transfer outcome could not be ascertained. Overall, 462 (20%) admissions resulted in patient death either on the SLH medical wards or following transfer to another ward or facility.

In total, while HIV‐positive patients accounted for 42.4% of admissions, they accounted for 47.2% of deaths. Admissions of HIV‐positive patients were 1.2 (95% CI: 1.0 to 1.5) times more likely to result in death compared with admissions of HIV‐negative patients. After adjusting for age, sex and co‐morbidities, HIV‐positive admissions were 1.6 (95% CI: 1.4 to 2.0) times more likely to result in death compared with HIV‐negative admissions. Of note, among admission of patients aged ≤60 years, HIV‐positive patients accounted for 79.7% of in‐hospital deaths and were 2.2 (95% CI: 1.6 to 3.0) times more likely to die compared to HIV‐negative patients in fully adjusted analysis. Median time‐to‐death during hospitalization was similar among HIV‐positive (four days, IQR 2 to 12) and HIV‐negative (five days, IQR 2 to 13) admissions. Hospitalization outcomes by HIV status are shown in Table 2.

**Table 2:** Hospitalization outcome per admission stratified by final HIV status^a^

Hospitalization outcome	HIV+ (n = 983)	HIV− (n = 1086)	HIV unknown (n = 247)	Total (n = 2316)
Died during admission	196 (19.9)	178 (16.4)	46 (18.6)	420 (18.1)
Discharged alive	724 (73.7)	834 (76.8)	191 (77.3)	1749 (75.5)
Left against medical advice	7 (0.71)	7 (0.64)	3 (1.2)	17 (0.7)
Transferred	56 (5.7)	67 (6.2)	7 (2.8)	130 (5.6)
Transfer destination				
Other ward in SLH	16 (28.6)	20 (29.9)	3 (42.9)	39 (30.0)
Tertiary centre (public)	35 (62.5)	40 (59.7)	4 (57.1)	79 (60.8)
Tertiary centre (private)	1 (1.8)	6 (9.0)	0 (0.0)	7 (5.4)
Tertiary centre (South Africa)	1 (1.8)	0 (0.0)	0 (0.0)	1 (0.77)
Other	3 (5.4)	1 (1.5)	0 (0.0)	4 (3.1)
Outcome after transfer				
Discharged alive	34 (60.7)	48 (71.6)	5 (71.4)	87 (66.9)
Dead	22 (39.3)	18 (26.9)	2 (28.6)	42 (32.3)
Unknown	0 (0.0)	1 (1.5)	0 (0.0)	1 (0.77)
Total known deaths	218 (22.2)	196 (18.0)	48 (19.4)	462 (20.0)

SLH, Scottish Livingstone Hospital.

a Data presented as n (%).

### 3.6 Most common diagnoses among admissions and mortality cases by HIV status

The most common diagnoses (e.g. per final diagnosis at discharge, death or transfer) among HIV‐positive admissions were tuberculosis (27.5%), bacterial pneumonia (9.6%), gastroenteritis (8.0%) and anaemia (5.3%). The most common diagnoses among HIV‐negative admissions were heart failure (9.2%), diabetes (8.7%), cerebrovascular event (8.7%) and tuberculosis (8.2%). Among mortality cases of patients with a final HIV‐positive status the most common diagnoses were tuberculosis (44.5%), bacterial pneumonia (6.4%), kidney disease (6.4%), Pneumocystis jiroveci pneumonia (6.0%) and cryptococcal meningitis (5.5%). Among mortality cases of patients with a final HIV‐negative status the most common diagnoses were tuberculosis (19.4%), cerebrovascular event (13.3%), solid malignancy (12.2%), heart failure (10.2%) and infection or sepsis not otherwise specified (10.2%).

Clinical or laboratory diagnosis of tuberculosis accounted for a higher proportion of admissions (27.5% vs. 8.2%, *p* < 0.0001) and a higher proportion of deaths (44.5% vs. 19.4%, *p* < 0.0001) among HIV‐positive admissions compared with HIV‐negative admissions respectively. Of note, among all patients, the majority of diagnoses of both pulmonary and extra pulmonary tuberculosis were made based on clinical criteria. Of the 283 patients diagnosed with pulmonary tuberculosis (with or without extra pulmonary disease), sputum AFB smear results where available for only 75 patients, 13 of which (17.3%) were sputum positive and sputum GeneXpert results were available for 39 patients, 15 of which (38.5%) were sputum positive. TB culture results were not available for any patient. In most cases unavailability of sputum AFB smear, GeneXpert and TB culture results was due to stock‐outs of lab reagents, equipment breakdown or patient inability to produce sputum. Table 3 lists the 10 most common diagnoses among admissions with known final HIV status, the observed associated mortality by HIV status and the corresponding crude and adjusted risk ratios for mortality among HIV‐positive versus HIV‐negative admissions. Admissions of HIV‐positive patients for gastroenteritis (aRR 11.8, 95% CI 1.5 to 96.3) and diabetes (aRR 6.6, 95% 1.2 to 36.6) were more likely to result in death when compared to admissions of HIV‐negative patients for the same conditions, after adjustment for age and sex (however these estimates are based on small numbers). Among patients admitted for any of the remaining eight most common diagnoses no significant differences in the likelihood of death were observed between HIV‐positive and HIV‐negative patients.

**Table 3:** Ten most common diagnoses and associated mortality stratified by HIV status among admissions with known final HIV status

Diagnosis	HIV+ (n = 983)		HIV− (n = 1086)		Crude RR (95% CI)	Adj RR^a^ (95% CI)
	n total	n (%) mortality	n total	n (%) mortality		
Tuberculosis	270	97 (35.9)	89	38 (42.7)	0.84 (0.63 to 1.1)	1.1 (0.82 to 1.5)
Bacterial pneumonia	94	14 (14.9)	87	19 (21.8)	0.68 (0.36 to 1.3)	1.1 (0.59 to 2.2)
Heart failure	27	4 (14.8)	100	20 (20.0)	0.74 (0.29 to 1.9)	0.84 (0.28 to 2.5)
Gastroenteritis	79	9 (11.4)	46	1 (2.2)	5.2 (0.68 to 40.2)	11.8 (1.5 to 96.3)
Diabetes	27	3 (11.1)	95	2 (2.1)	5.3 (0.93 to 30.1)	6.6 (1.2 to 36.6)
Cerebrovascular event	15	1 (6.7)	94	26 (27.7)	0.24 (0.04 to 1.7)	0.28 (0.04 to 2.0)
Suicide attempt	31	0 (0.0)	53	0 (0.0)	–	–
Anaemia	52	1 (1.9)	32	0 (0.0)	–	–
Infection or sepsis NOS	20	6 (30.0)	52	20 (38.5)	0.78 (0.38 to 1.6)	1.9 (0.65 to 5.5)
Solid malignancy	15	3 (20.0)	55	24 (43.6)	0.46 (0.17 to 1.2)	0.56 (0.19 to 1.6)
Other^b^	353	80 (22.7)	383	46 (12.0)	1.9 (1.35 to 2.64)	2.35 (1.7 to 3.3)

NOS, not otherwise specified.

a Risk of death of HIV positive relative to HIV negative, adjusted for age and sex

b Any diagnosis other than specified above.

### 3.7 Risk factors for in‐hospital mortality among HIV‐positive patients

Risk factors for mortality among HIV‐positive patient admissions are shown in Table 4, and included older age, male sex, low CD4 count, detectable HIV RNA and not being on ART. Among HIV positive admissions, after adjustment for age, sex and co‐morbidities, male sex was associated with1.4 (95% CI: 1.0 to 1.7) times increased risk of death; admission with a CD4 count <200 cells/mm^3^ was 1.5 (95% CI: 1.2 to 2.0) times more likely to result in death compared to admission with a CD4 count ≥200 cells/mm^3^; and admission with a detectable HIV RNA viral load of >400 copies/mL was 1.5 (95% CI: 1.1 to 2.1) times more likely to result in death compared to admission with an undetectable HIV RNA viral load (<400 copies/mL).The risk of mortality during admissions of HIV‐positive patients who were not on ART was significantly higher compared with admissions of HIV‐positive patients who were on ART prior to admission (29.8% vs. 19.5%, *p* < 0.001). After adjusting for age, sex and co‐morbidities being on ART prior to admission conferred a 31% (95% CI: 12.0 to 47.0%) reduced risk of death compared to not being on ART. Overall mortality among HIV‐positive admissions who were on ART prior to admission did not differ significantly from that of HIV‐negative admissions (19.5% vs. 18.0%, *p* = 0.44). However, after adjusting for age, the risk of mortality among HIV‐positive patients on ART was significantly higher when compared to HIV‐negative patients (aRR 1.6, CI: 1.3 to 1.9).

**Table 4:** Risk factors for death among admissions of patients with known final HIV‐positive status^a^

Risk factor	Alive (n = 765)	Dead (n = 218)	RR (95% CI)	Adj RR^b^ (95% CI)
Age (years)	42 (33 to 52)	43 (35 to 55)	1.01 (1.00 to 1.02)	1.01 (1.00 to 1.02)
Sex				
Male	353 (46.1)	125 (57.3)	1.42 (1.11 to 1.80)	1.37 (1.08 to 1.74)
Female	412 (53.9)	93 (42.7)		
Co‐morbidities^c^				
Any	282 (36.9)	71 (32.6)	0.86 (0.67, 1.11)	0.80 (0.61 to 1.04)
None	483 (63.1)	147 (67.4)		
Most recent CD4 count (cells/mm^3^)				
<200	296 (38.7)	108 (49.5)	1.59 (1.24 to 2.05)	1.51 (1.16 to 1.96)
≥200	411 (53.7)	83 (38.1)		
Missing	58 (7.6)	27 (12.4)		
HIV‐RNA viral load (copies/mL)				
≥400	108 (14.1)	37 (17.0)	1.43 (1.04 to 1.97)	1.52 (1.11 to 2.09)
<400	437 (57.1)	95 (43.6)		
Missing	220 (28.8)	86 (39.4)		
ART status at admission				
On ART	526 (68.8)	127 (58.3)	0.65 (0.51 to 0.83)	0.69 (0.53 to 0.88)
Not on ART	174 (22.8)	74 (33.9)		
Missing	65 (8.5)	17 (7.8)		
HIV status at admission				
Unknown HIV status	63 (8.2)	16 (7.3)	0.91 (0.57 to 1.43)	0.89 (0.56 to 1.40)
Known HIV‐positive status	702 (97.8)	202 (92.7)		

ART, anti‐retroviral therapy.

a Data presented as n (%) or mean ± standard deviation

b adjusted for age, sex and co‐morbidities

c Patients were considered to have a known co‐morbidity if the medical recorded indicated a history of hypertension, diabetes, heart disease, lung disease, previous episodes of tuberculosis or any other chronic disease (see Table 1 for listed co‐morbidities stratified by HIV status).

### 3.8 Comparison with 2011 to 2012 surveillance

We compared our results with those of similar surveillance conducted at SLH over a 6‐month period between November 2011 and April 2012 14.During the current surveillance period, overall final HIV prevalence was lower (42.4% vs. 47.6% of admissions, *p* < 0.01) and the proportion of patients with unknown HIV status on admission declined (36.1 vs. 41.2% *p* < 0.01) as did the proportion of patients whose HIV status was unknown at discharge or death (10.7% vs. 27.7% *p* < 0.0001). Among HIV‐positive admissions in the current surveillance period, ART‐coverage was higher (72.2% vs. 56.2%, *p* < 0.0001), mortality was similar (22.2% vs. 22.7%, *p* = 0.93), tuberculosis diagnoses increased (27.5% vs. 20.1%, *p* < 0.01) and tuberculosis associated mortality was higher (35.9% vs. 24.7%, *p* = 0.05). Among all admissions, in the current surveillance period tuberculosis diagnoses increased (15.7% vs. 12.9%, *p* = 0.04) and tuberculosis associated mortality was higher (37.6% vs. 24.0%, *p* < 0.01). Table 5 shows a summary comparison of the current surveillance data with data from 2011 to 2012.

**Table 5:** Comparison of 2015 to 2017 and 2011 to 2012 surveillance data at Scottish Livingstone Hospital, Botswana^a^

	2015 to 2017 (24 months data)	2011 to 2012 (6 months data)	*p*‐value
Among all admissions	n = 2316	n = 972	
Age (years)	52.3 ± 22.0	47.8 ± 20.4	<0.0001
Female sex	1237 (53.4)	518 (53.3)	0.99
Final HIV positive status	983 (42.4)	463 (47.6)	0.006
Tuberculosis diagnosis	364 (15.7)	125(12.9)	0.04
Died	462 (19.9)	172 (17.7)	0.69
Died, tuberculosis diagnosis^b^	137 (37.6)	30 (24.0)	0.006
Among HIV‐positive admissions (final status)	n = 983	n = 463	
Age (years)	43.5 ± 14.4	41 ± 12	0.001
Female sex	505 (51.4)	247 (53.3)	0.79
On ART at admission^c^	653 (72.5)	240 (56.2)	<0.0001
CD4 count≥200 cells/mm^3^ b	494 (55.0)	157 (44.6)	0.0009
HIV‐RNA viral load <400 copies/mL^d^	532 (78.6)	126 (80.3)	0.77
Tuberculosis diagnosis	270(27.5)	93(20.1)	0.003
Died	218 (22.2)	105 (22.7)	0.93
Died, tuberculosis diagnosis^e^	97 (35.9)	23 (24.7)	0.048

a Data presented as mean ± SD or n(%)

b n (%) among admissions with diagnosis of tuberculosis

c n (%) among known HIV‐positive at admission (2015 to 2017, n = 904; 2011 to 2012, n = 427)

d n (%) among admissions with available CD4 count from prior to or during admission (2015 to 2017, n = 898; 2011 to 2011, n = 352)

e n (%) among admissions with available HIV‐RNA viral load from prior to or during admission (2015 to 2017, n = 677; 2011 to 2012, n = 157).

## 4 Discussion

This is the largest study to date examining the impact of HIV on medical inpatient morbidity and mortality in the ART era in Botswana. Our findings demonstrate an ongoing high burden of HIV‐related inpatient mortality, particularly from tuberculosis. HIV‐positive patients were disproportionately represented among hospital admissions and deaths, and admissions of HIV‐positive patients were more likely to result in death compared with those of HIV‐negative patients. Mortality was particularly high among HIV‐positive males and among HIV‐positive patients who were not on ART, and, of importance, HIV was also associated with a significantly younger age of both admission and death. Tuberculosis accounted for 44.5% of all mortality among HIV‐positive patients, and a significantly higher proportion of mortality compared with HIV‐negative admissions.

The introduction of highly active ART in developed countries in the mid 1990's led to significant decline in hospital admissions for AIDS‐defining conditions and opportunistic infections 15, 16, 17, 18. However, a recent systematic review and meta‐analysis of hospital admissions among people living with HIV globally showed that AIDS‐related illnesses and bacterial infections continue to be the most common causes of hospital admissions and mortality among HIV‐positive children and adults in all geographical regions 19. Few ART‐era studies from the African region have previously reported on rates of ART‐coverage and mortality among HIV‐positive adult medical admissions. The HIV‐related medical inpatient mortality rate of 22% on our wards was similar to that from hospital settings in Malawi (24%, 25%) where a comparable ART‐coverage rate (72%) was reported 3, 4 and lower compared to a multi‐centre study from West Africa which reported 38% mortality in the setting of lower (48%) ART‐coverage 5. Widely divergent HIV‐related inpatient mortality rates of 17% and 30% have been reported from settings in Kenya and DRC respectively, where a relatively high proportion of patients were on ART at the time of admission 20. At the same time another study, from a district hospital in South Africa, reported lower rates of mortality (13.3%) despite lower ART‐coverage (45%) and higher rates of tuberculosis (33.5%) 21. As in our study, tuberculosis was reported to be the most common cause of hospitalization and death among HIV‐positive patients in all these settings. These findings suggest that beyond ART‐coverage, hospitalization outcomes among HIV‐positive patients are likely influenced by other factors including morbid and co‐morbid illnesses, the quality of pre‐hospitalization HIV‐care, early identification and management of HIV treatment failure and tuberculosis, available healthcare resources and the quality of inpatient care.

We compared our findings with similar surveillance conducted at SLH in 2011 to 2012. These comparisons revealed an improvement in HIV testing both pre‐ and post‐admission, and a significant decline in HIV prevalence, but a surprising stability in mortality among hospitalized HIV‐positive patients despite increased ART use. Strikingly, tuberculosis‐associated mortality among HIV‐positive patients increased by 11% between the two surveillance periods. Our finding of stable inpatient mortality by study era despite national population‐level estimates of an overall decline in AIDS‐related mortality 11 and HIV/TB‐related deaths 22 during the same period, suggests that ART availability has in itself been insufficient to curb the impact of HIV on in‐hospital mortality, especially from tuberculosis. Further study is needed to understand whether regional TB‐trends, the coverage of ART in specific regions and populations, or the timing of a patient's HIV diagnosis and ART initiation in relation to admission, may impact these findings.

Our study's strengths were its complete accounting for all patient outcomes during a 2‐year period, including outcomes following transfer. Limitations were primarily related to our reliance on inpatient chart extraction, rather than patient interviews and outpatient clinical records. Results of pre‐admission HIV testing, CD4 cell counts and viral loads were extracted from the inpatient chart and from the national electronic medical record which captures some but not all viral and immunologic testing occurring in the outpatient setting. The number of patients classified as having unknown HIV status on admission may have been overestimated if results of community‐based HIV testing were not recorded in the inpatient chart or the electronic medical record. Many HIV‐positive patients had CD4 counts and viral loads checked during their admission but in the absence of inpatient results, patients' most recent pre‐admission results were used in our analysis and may not have reflected CD4 and viral load levels at the time of admission. We were also unable to ascertain prophylactic use of sulfamethoxazole‐trimethoprim prior to hospitalization which may impact morbidity and mortality among hospitalized HIV‐positive patients. Limitations in available diagnostics and documentation and unavailability of autopsy data may have also led to inaccuracies in classification of diagnoses. While tuberculosis was found to be a leading cause of admission and mortality, most diagnoses of tuberculosis were made based on clinical criteria and microbiological confirmation was available for only a minority of patients. Given the non‐specific nature of the clinical signs and symptoms of tuberculosis, some patients who were classified as tuberculosis cases, especially among those who suffered mortality (and therefore may have not responded to anti‐tuberculosis therapy) may have had different underlying diagnoses which could have been missed. Limitations in the accuracy and availability of tuberculosis diagnostic modalities present challenges to the clinical management of suspected TB cases in both high‐resource and resource‐limited settings. Previous studies in similar settings in Africa reported low rates of microbiological confirmation of suspected tuberculosis even when modalities such as TB culture are available 6, 8. Reported incidence risks of tuberculosis in this study nevertheless are comparable to those reported in other settings with similar burdens of HIV and tuberculosis. Finally, this study was designed to describe mortality of patients after admission to the medical department in our hospital and did not capture deaths which may have occurred prior to hospital presentation and in the emergency department prior to admission to the medical wards, nor can our results be used to infer mortality rates at a population level.

## 5 Conclusions

Notwithstanding significant gains in the fight against HIV/AIDS and the high ART‐coverage rates achieved in Botswana, we found that HIV‐positive patients continue to be disproportionately represented among hospital admissions and deaths. Tuberculosis continues to be the leading presumed cause of morbidity and mortality among HIV‐positive patients. Our results are consistent with findings from other settings in sub‐Saharan Africa demonstrating ongoing high rates of HIV‐positive admissions and associated mortality (especially from tuberculosis) despite increased availability of ART 3, 4, 5, 6, 7, 8, 20, 21, and contrast with experience from Europe and North America where HIV has become a condition largely managed in the outpatient setting 15, 16, 17, 18. These differences are likely explained by health system resources, differences in tuberculosis prevalence, different spectrum of opportunistic infections and socio‐economic factors which disadvantage patients in developing‐country settings. The high proportion of patients in our study who had not been previously tested for HIV, and the concentration of excess mortality among HIV‐positive patients who are not on ART suggest that concentrated efforts to identify HIV‐positive patients and initiate ART early in the outpatient setting may be essential to reduce inpatient mortality and improve patient outcomes in our setting. In addition, expansion of case‐finding efforts, targeting suspected tuberculosis cases for early diagnosis and treatment and addressing the socio‐economic factors that drive the tuberculosis epidemic may help curb the devastating impact this infection continues to have on morbidity and mortality in Botswana and similar developing‐country settings throughout the world.

## Competing interests

All authors: no reported competing interests.

## Authors' contributions

TB, NT, RZ, KK, SL and RS contributed to study conceptualization. TB oversaw the study. RS obtained funding. TB, NT, RZ, SL and RS contributed to study design. TB and NT designed the data collection tool. TB and PM oversaw data collection. DN conducted the main statistical analysis. TB contributed to statistical analysis. TB wrote the initial draft of the manuscript. TB, DN, NT, RZ, KK, MP, MM, JM, SL and RS reviewed and edited the manuscript. All authors have read and approved the final manuscript.

## Funding

This work was partially supported by funds from the Center for AIDS Research (CFAR) at the National Institute of Health, US.
